# Differential effects of structurally different lysophosphatidylethanolamine species on proliferation and differentiation in pre-osteoblast MC3T3-E1 cells

**DOI:** 10.1038/s41598-024-84176-8

**Published:** 2025-01-02

**Authors:** Fumiaki Makiyama, Shiori Kawase, Aoi William Omi, Yusuke Tanikawa, Taishi Kotani, Teruki Shirayama, Naoyuki Nishimura, Taiga Kurihara, Naoto Saito, Jun Takahashi, Takeshi Uemura

**Affiliations:** 1 Department of Orthopedic Surgery, Shinshu University School of Medicine, Nagano, 390-8621 Japan; 2 Department of Biomedical Engineering, Graduate School of Medicine, Science and Technology, Shinshu University, Nagano, 390-8621 Japan; 3 Division of Gene Research, Research Center for Advanced Science and Technology, Shinshu University, Nagano, 390-8621 Japan; 4 Department of Biomedical Engineering, Graduate School of Science and Technology, Shinshu University, Nagano, 390-8621 Japan; 5 Institute for Biomedical Sciences, Interdisciplinary Cluster for Cutting Edge Research, Shinshu University, Nagano, 390-8621 Japan; 6 Division of Microbiology and Molecular Cell Biology, Nihon Pharmaceutical University, Saitama, 362- 0806 Japan; 7 Division of Physiology, Faculty of Medicine, Saga University, Saga, 849-8501 Japan

**Keywords:** Lysophosphatidylethanolamine, Pre-osteoblast cell, Proliferation, Differentiation, G protein-coupled receptors, Biochemistry, Cell biology

## Abstract

Lysophosphatidylethanolamine (LPE) is a bioactive lipid mediator involved in diverse cellular functions. In this study, we investigated the effects of three LPE species, 1-palmitoyl LPE (16:0 LPE), 1-stearoyl LPE (18:0 LPE), and 1-oleoyl LPE (18:1 LPE) on pre-osteoblast MC3T3-E1 cells. All LPE species stimulated cell proliferation and activated the mitogen-activated protein kinase (MAPK)/extracellular signal-regulated kinase (ERK) 1/2. MAPK/ERK1/2 activation by 16:0 LPE and 18:1 LPE was inhibited by the Gq/11 inhibitor YM-254890, while activation by 18:0 LPE was blocked by the Gi/o inhibitor pertussis toxin. Intracellular Ca^2+^ transients were triggered by 16:0 LPE and 18:1 LPE but not by 18:0 LPE, with YM-254890 suppressing these responses. These results suggest that 16:0 and 18:1 LPE act via Gq/11-coupled G protein coupled receptors (GPCRs), and 18:0 LPE acts via Gi/o-coupled GPCRs. Furthermore, receptor desensitization experiments suggested that each LPE acts through distinct GPCRs. Interestingly, 18:0 LPE suppressed osteogenic differentiation, reducing mineralization, alkaline phosphatase activity, and osteogenic gene expression, whereas 16:0 LPE and 18:1 LPE had no such effects. These results suggest the physiological significance of LPEs in bone formation and indicate that different LPE species and their receptors play distinctive roles in this process.

**Supplementary Information:**

The online version contains supplementary material available at 10.1038/s41598-024-84176-8.

## Introduction

Lysophospholipids are bioactive lipid mediators that play various roles in physiological and pathological conditions through their distinct G protein-coupled receptors (GPCRs)^1–3^. Lysophosphatidylethanolamine (LPE) is a lysophospholipid produced from phosphatidylethanolamine through the action of a phospholipase A-type enzyme^1^. LPE comprises a glycerol backbone with an ethanolamine head group, a phosphate group, and a single fatty acid chain. Like other lysophospholipids, multiple LPE species can be found in mammals; they vary in the length and saturation degree of their fatty acids^4^. LPEs are minor constituents of mammalian cell membranes^4^and are also detected in human plasma and cerebrospinal fluid from submicromolar to micromolar levels^5,6^.

LPEs exert diverse functions in a cell type-specific manner, as evidenced by several studies. For example, egg yolk LPE stimulates the differentiation and maturation of cultured mouse astrocytes^7^. 1-oleoyl LPE (18:1 LPE) promotes chemotactic migration and cellular invasion in human ovarian cancer SK-OV3 cells^8^. 2-arachidonyl LPE (20:4 LPE) inhibits lipopolysaccharide-induced M1 macrophage polarization in mouse peritoneal macrophages^9^. Our previous studies have demonstrated that 1-palmitoyl LPE (16:0 LPE), 1-stearoyl LPE (18:0 LPE), and 1-oleoyl LPE (18:1 LPE) stimulate neurite outgrowth, and 18:1 LPE also protects neurons against glutamate toxicity in cultured cortical neurons^10,11^. However, the physiological functions of LPE with multiple molecular species in various mammalian cells and organs remain largely unknown.

Osteoblast lineage cells are crucial in bone formation, a complex process involving cell proliferation, differentiation, and the formation of a mineralized bone matrix^12,13^. Various factors, including extracellular matrix components, cytokines, hormones, and growth factors, play a role in the osteogenic process^13–16^. The role of bioactive lysophospholipids in physiological and pathological processes during bone formation has attracted attention. Notably, lysophospholipids, such as lysophosphatidic acid (LPA) and sphingosine-1-phosphate, have been reported to play essential roles in bone formation^3,17^. Intriguingly, a recent study on the spatial mass spectrometry analysis of lipids in mouse joint tissues suggested that LPE may act in the growth plate region of the knee joint, linked to its function in bone growth and mineralization^18^. However, the role of LPEs in bone formation remains to be elucidated.

The pre-osteoblastic MC3T3-E1 cell line, derived from mouse calvaria, is a valuable model for studying osteoblast biology in vitro because of its capacity to differentiate and form calcified nodules resembling woven bone^19,20^. In this study, we used MC3T3-E1 cells to investigate the effects of different LPE species on bone formation. Our results suggest the importance of LPEs in bone formation and that different LPE species have different effects.

## Results

### LPEs promote MC3T3-E1 cell proliferation

**Fig. 1:**
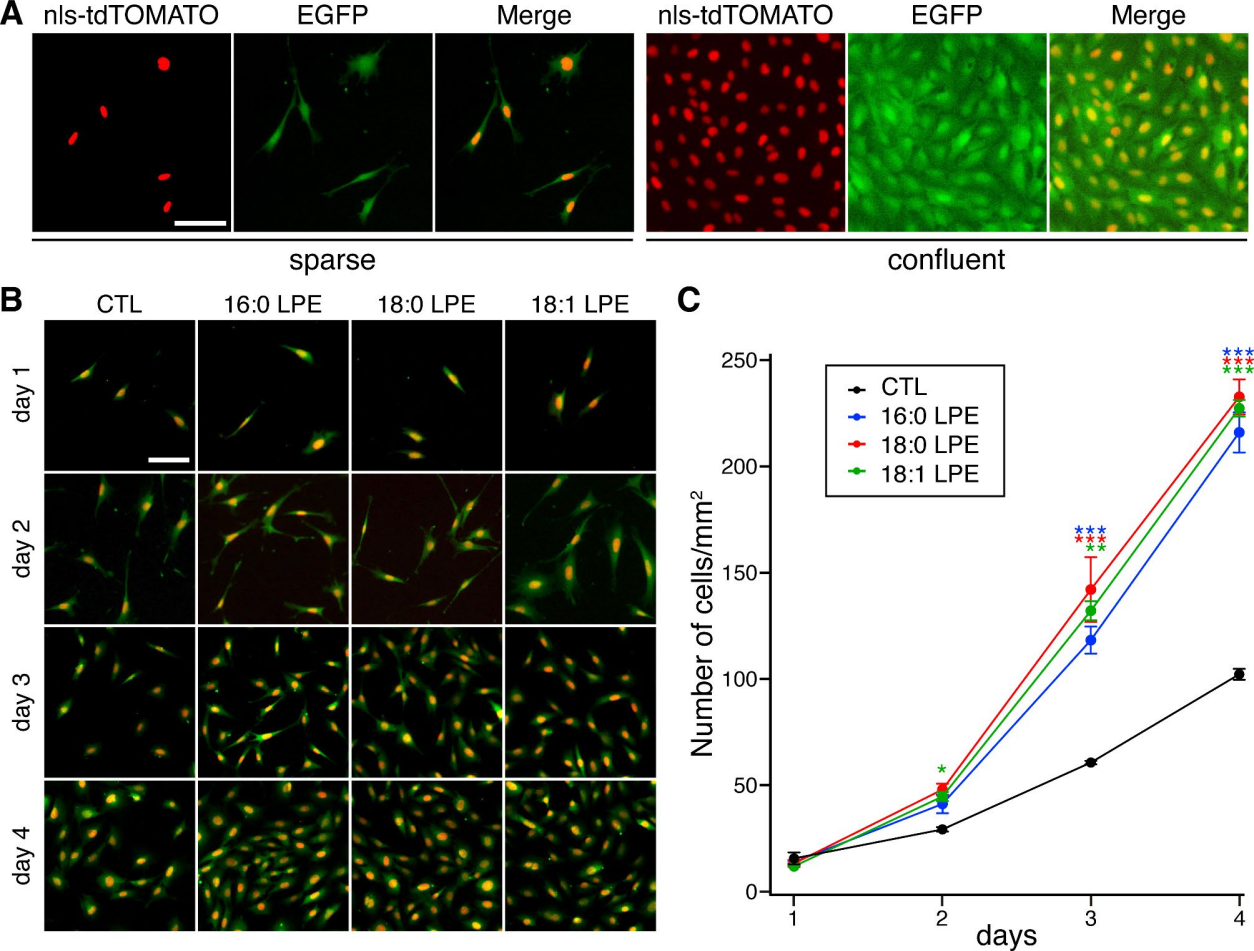
LPEs stimulate MC3T3-E1 cell growth. (**A**) MC3T3-E1 stable cell line expressing EGFP and NLS-tdTOMATO. Representative fluorescence images showing cells in sparse and confluent states. Images were merged to illustrate cell density and distribution. (**B**) Effects of LPEs on MC3T3-E1 cell proliferation. Cells were incubated with media containing 10 µM 16:0 LPE, 18:0 LPE, or 18:1 LPE. Merged images of EGFP and NLS-tdTOMATO fluorescence from days 1 to 4 are shown. (**C**) Quantification of the number of cells in (**B**). Data are presented as mean ± SEM (*n* = 4 cultures each). ***p < 0.001, **p < 0.01, and *p < 0.05 compared with the control; repeated measures two-way ANOVA followed by a post-hoc Tukey’s test. Scale bars, 50 μm.

We established an MC3T3-E1 cell line stably expressing enhanced green fluorescent protein (EGFP) and the tandem dimer Tomato fused to a nuclear localization signal (NLS-tdTOMATO) to observe MC3T3-E1 cells in a living state. In the established cell line, the EGFP signal was observed throughout the cell, whereas the tdTOMATO signal was localized to the nucleus (Fig. 1A). To examine the effects of LPE species on the MC3T3-E1 cell proliferation, MC3T3-E1 cells expressing EGFP and NLS-tdTOMATO were incubated with media containing 16:0 LPE, 18:0 LPE, or 18:1 LPE. Cell proliferation was observed in all groups from day 1 to day 4, with no obvious effects on the morphology of cells treated with LPEs (Fig. 1B). Notably, cells treated with 16:0 LPE, 18:0 LPE, and 18:1 LPE exhibited a significant increase in their number compared with the control culture, indicating that LPEs promote cell proliferation (Fig. 1B, C). No substantial differences in proliferation were observed among the application of 16:0 LPE, 18:0 LPE, and 18:1 LPE.

It has been reported that both the action and signaling of LPE are mediated through lysophosphatidic acid receptor 1 (LPA1), but this involvement varies by cell type, with some mediated by LPA1 and others using non-LPA receptors^8,21–24^. We investigated the potential involvement of LPA1 in the response to LPEs in MC3T3-E1 cells. The expression level of *Enpp2* mRNA, which encodes autotaxin, an enzyme responsible for converting lysophospholipids into LPA, was extremely low or nearly undetectable in MC3T3-E1 cells. The growth stimulation induced by 16:0 LPE, 18:0 LPE, and 18:1 LPE in MC3T3-E1 cells was unaffected by the LPA1 antagonist AM095, whereas the growth stimulation by 1-oleoyl LPA (18:1 LPA) was completely suppressed by AM095 (Supplementary Fig. 1).

## LPE species activate mitogen-activated protein kinase via distinct GPCRs in MC3T3-E1 cells

**Fig. 2:**
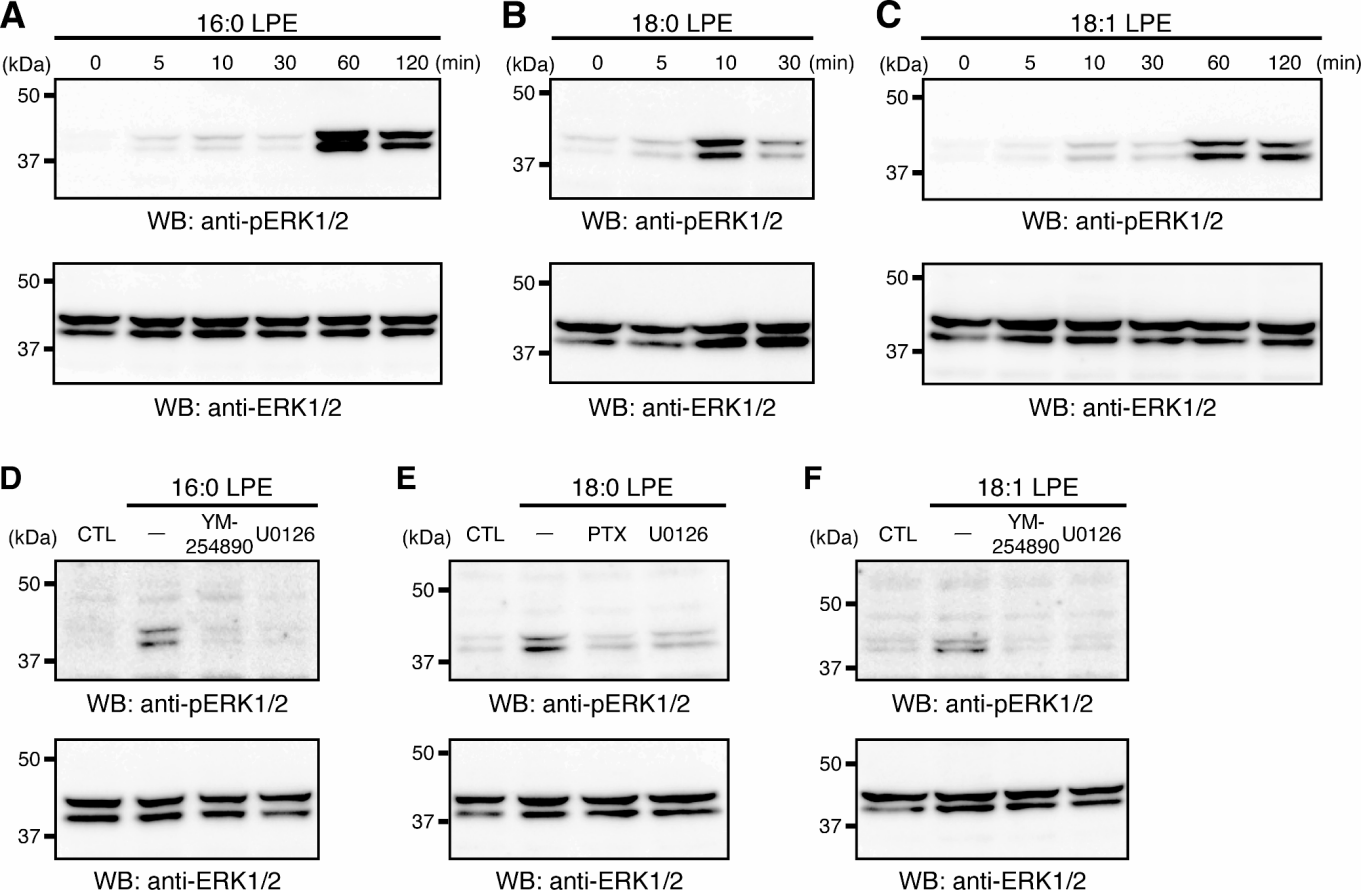
LPEs activate the MAPK/ERK1/2 in MC3T3-E1 cells via distinct GPCR types. (**A**–**C**) MAPK/ERK1/2 activation by 16:0 LPE, 18:0 LPE, and 18:1 LPE in MC3T3-E1 cells. The cells were incubated with 10 μM 16:0 LPE (**A**), 18:0 LPE (**B**), or 18:1 LPE (**C**). After incubation for the indicated times, cells were lysed and analyzed by western blotting using antibodies against ERK1/2 and pERK1/2 proteins. (**D**) Effects of Gq/11 inhibitor YM-254890 on ERK1/2 phosphorylation induced by 16:0 LPE. Cell lysates were prepared 60 min after the incubation with 10 μM 16:0 LPE. (**E**) Effects of Gi/o inhibitor PTX on ERK1/2 phosphorylation induced by 10 μM 18:0 LPE. Cell lysates were prepared 10 min after the incubation with 18:0 LPE. (**F**) Effects of Gq/11 inhibitor YM-254890 on ERK1/2 phosphorylation induced by 10 μM 18:1 LPE. Cell lysates were prepared 60 min after the incubation with 18:1 LPE. MAPK/ERK1/2 inhibitor U0126 inhibited ERK1/2 phosphorylation induced by LPEs (**D**–**F**). The original images are shown in Supplementary Fig. 4.

Our previous studies demonstrated that 16:0 LPE, 18:0 LPE, and 18:1 LPE activate mitogen-activated protein kinase (MAPK)/extracellular signal-regulated kinase (ERK) 1/2 via distinct GPCRs in cultured cortical neurons^10,11^. Thus, we examined whether the MAPK/ERK1/2 signal cascade was activated in LPE-treated MC3T3-E1 cells. In the control culture, anti-phospho-ERK1/2 antibody detected bands corresponding to the size of phosphorylated ERK1/2, and incubating with 16:0 LPE, 18:0 LPE, or 18:1 LPE increased these signals (Fig. 2). ERK1/2 phosphorylation peaked 60 min after stimulation with 16:0 LPE and 18:1 LPE, whereas treatment with 18:0 LPE resulted in maximum phosphorylation at 10 min post-stimulation (Fig. 2A–C). Furthermore, 16:0 LPE- and 18:1 LPE-induced ERK1/2 phosphorylation was suppressed by the application of Gq/11 inhibitor YM-254890, whereas 18:0 LPE-induced ERK1/2 phosphorylation was suppressed by the application of Gi/o inhibitor pertussis toxin (PTX). All LPE-induced ERK1/2 phosphorylation was inhibited by MAPK inhibitor U0126 (Fig. 2D–F). These results suggested that 16:0 LPE and 18:1 LPE activate the Gq/11-coupled receptor, whereas 18:0 LPE activates the Gi/o-coupled receptor in MC3T3-E1 cells.

## Differential effects of structurally distinct LPE species on intracellular Ca^2+^ responses in MC3T3-E1 cells

**Fig. 3:**
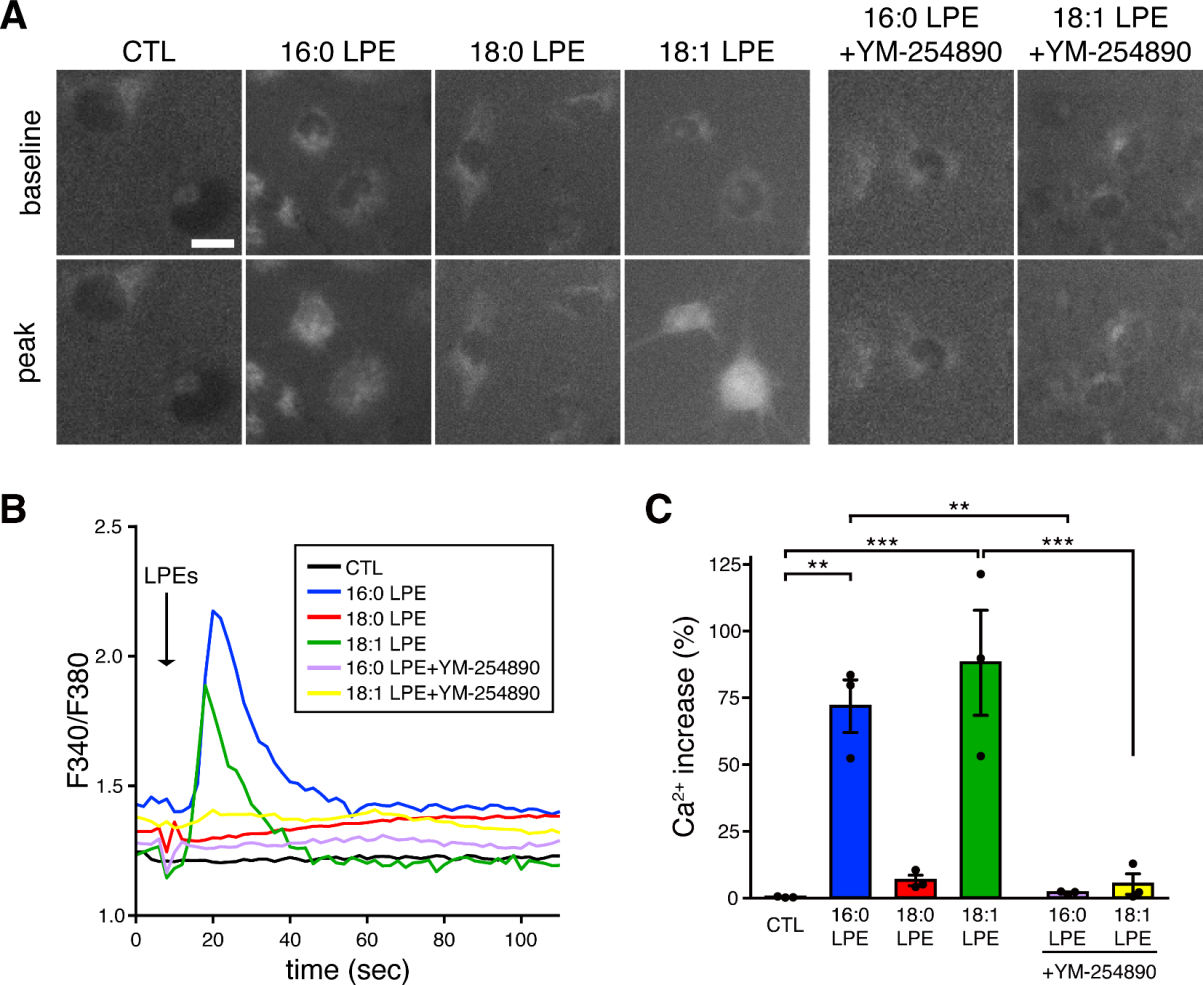
Differential effects of LPE species on Ca^2+^ response in MC3T3-E1 cells. (**A**) Effects of 16:0 LPE, 18:0 LPE, and 18:1 LPE on [Ca^2+^]i response in MC3T3-E1 cells. The cells were stimulated with 10 µM 16:0 LPE, 18:0 LPE, or 18:1 LPE in the presence or absence of the Gq/11 inhibitor YM-254890. [Ca^2+^]i levels were monitored by Fura 2-AM. Images of Fura 2 fluorescence ratio (F340/F380) are shown. CTL; DMSO vehicle-treated control. Scale bar, 20 μm. (**B**) Representative traces of changes in the F340/F380 ratio, an indicator of [Ca²⁺]i, are shown. (**C**) Summary of results in (**B**). Percentages of maximum increase in [Ca²⁺]i were quantified. Data are presented as mean ± SEM (*n* = 3). ***p < 0.001 and **p < 0.01; one-way ANOVA, followed by a post-hoc Tukey’s test.

Next, we examined whether the activation of different GPCRs by specific LPE species leads to distinct intracellular Ca^2+^ responses in MC3T3-E1 cells. To analyze the intracellular Ca^2+^ concentration ([Ca^2+^]i), cells were loaded with Fura 2-AM and stimulated with 16:0 LPE, 18:0 LPE, or 18:1 LPE. When 16:0 LPE and 18:1 LPE were applied to the cultures, MC3T3-E1 cells rapidly exhibited transient elevations in [Ca^2+^]i levels (Fig. 3A, B). In contrast, 18:0 LPE had little effect on the [Ca^2+^]i response. Peak amplitudes of [Ca^2+^]i in 16:0 LPE- and 18:1 LPE-treated cells were significantly higher than those in the control (Fig. 3C). The increase in [Ca^2+^]i induced by 16:0 LPE and 18:1 LPE was inhibited by the application of YM-254890 (Fig. 3A–C). No significant difference was observed between the responses to 16:0 LPE and 18:1 LPE. These results suggest that the effects on [Ca^2+^]i vary depending on the LPE species.

**Fig. 4:**
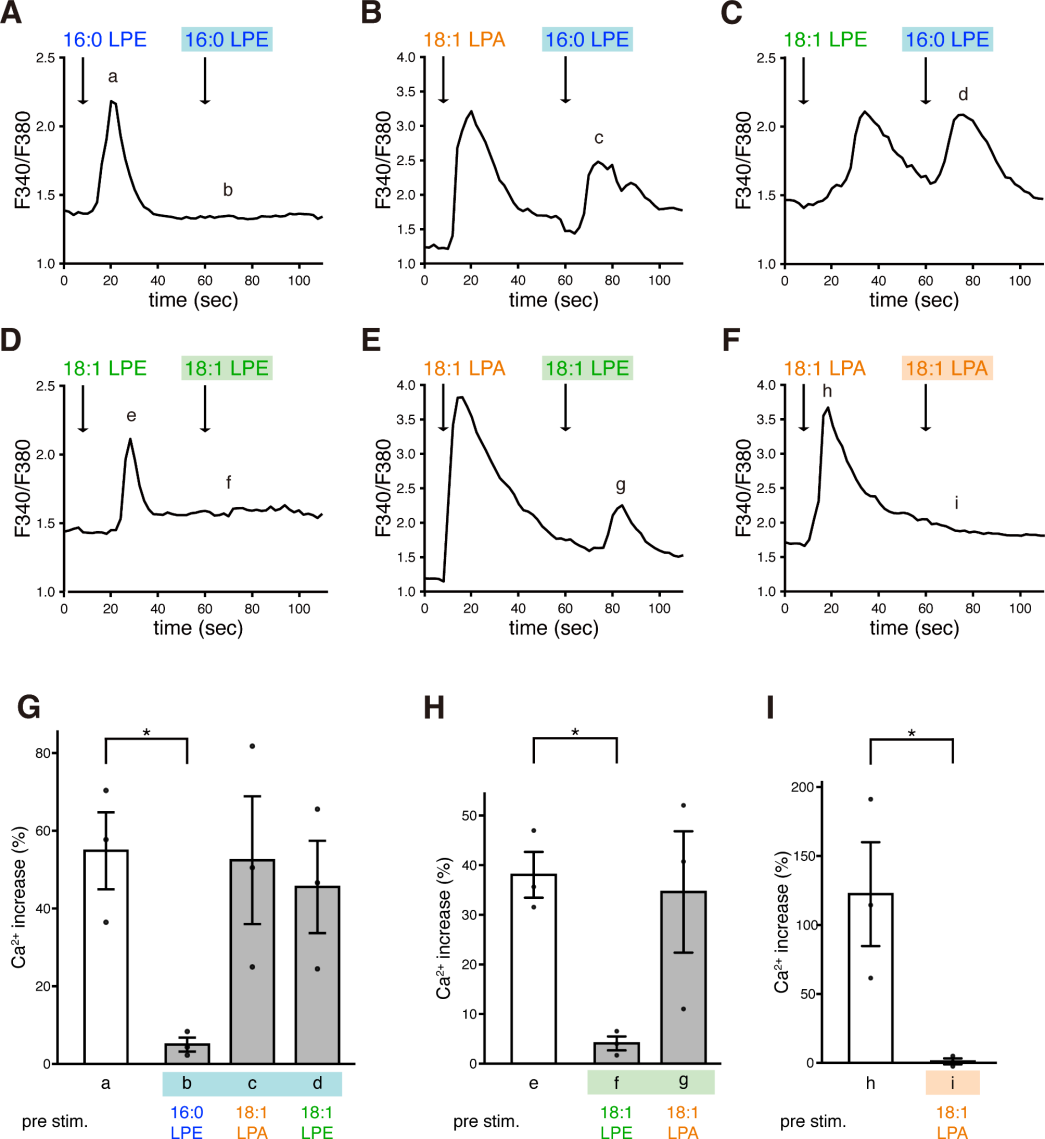
Desensitization of LPE-induced Ca^2+^ responses in MC3T3-E1 cells. (**A**–**C**) Effect of pre-stimulation with 16:0 LPE (A), 18:1 LPA (**B**), or 18:1 LPE (**C**) on Ca²⁺ response induced by 16:0 LPE. Changes in the F340/F380 ratio, an indicator of [Ca²⁺]i, are shown. (**D**, **E**) Effect of pre-stimulation with 18:1 LPE (**D**) or 18:1 LPA (E) on the Ca²⁺ response induced by 18:1 LPE. (F) The effect of pre-stimulation with 18:1 LPA on the Ca²⁺ response induced by 18:1 LPA. (**G**) Quantification of the percentage of maximum increase in [Ca²⁺]i induced by 16:0 LPE in (A–C). (**H**) Quantification of the percentage of maximum increase in [Ca²⁺]i induced by 18:1 LPE in (D, E). (**I**) Quantification of the percentage of maximum increase in [Ca²⁺]i induced by 18:1 LPA in (**F**). Labels a–i in (**G**–**I**) indicate the specific points analyzed within (**A**–**F**). In all experiments, 16:0 LPE, 18:0 LPE, 18:1 LPE, and 18:1 LPA were added at a concentration of 10 µM. Data are presented as mean ± SEM (*n* = 3). *p < 0.05; one-way ANOVA, followed by Dunnett’s test in (G, H) and Student’s t-test in (**I**).

We further examined the desensitization of LPE-induced Ca²⁺ responses, as repeated GPCR stimulation is known to induce desensitization^25^. When MC3T3-E1 cells were stimulated with 16:0 LPE, a significant increase in intracellular [Ca²⁺] levels was observed. However, subsequent stimulation with 16:0 LPE failed to elicit a [Ca²⁺]i response, indicating homologous desensitization (Fig. 4A, G). In contrast, pre-stimulation with 18:1 LPA or 18:1 LPE did not affect the [Ca²⁺]i response induced by 16:0 LPE during subsequent stimulation (Fig. 4B, C, G). Pre-stimulation with 18:1 LPE suppressed the [Ca²⁺]i elevation induced by subsequent 18:1 LPE stimulation, whereas 18:1 LPA pre-stimulation had no such inhibitory effect (Fig. 4D, E, H). Additionally, 18:1 LPA demonstrated homologous desensitization (Figs. 4F, I). Finally, pre-stimulation with 18:0 LPE did not influence the [Ca²⁺]i responses induced by either 16:0 LPE or 18:1 LPE (Supplementary Fig. 2). These results suggested that 16:0 LPE and 18:1 LPE mediate Ca²⁺ responses through different receptors, other than LPA receptors.

## Differential effects of structurally distinct LPE species on osteogenic differentiation in MC3T3-E1 cells

**Fig. 5:**
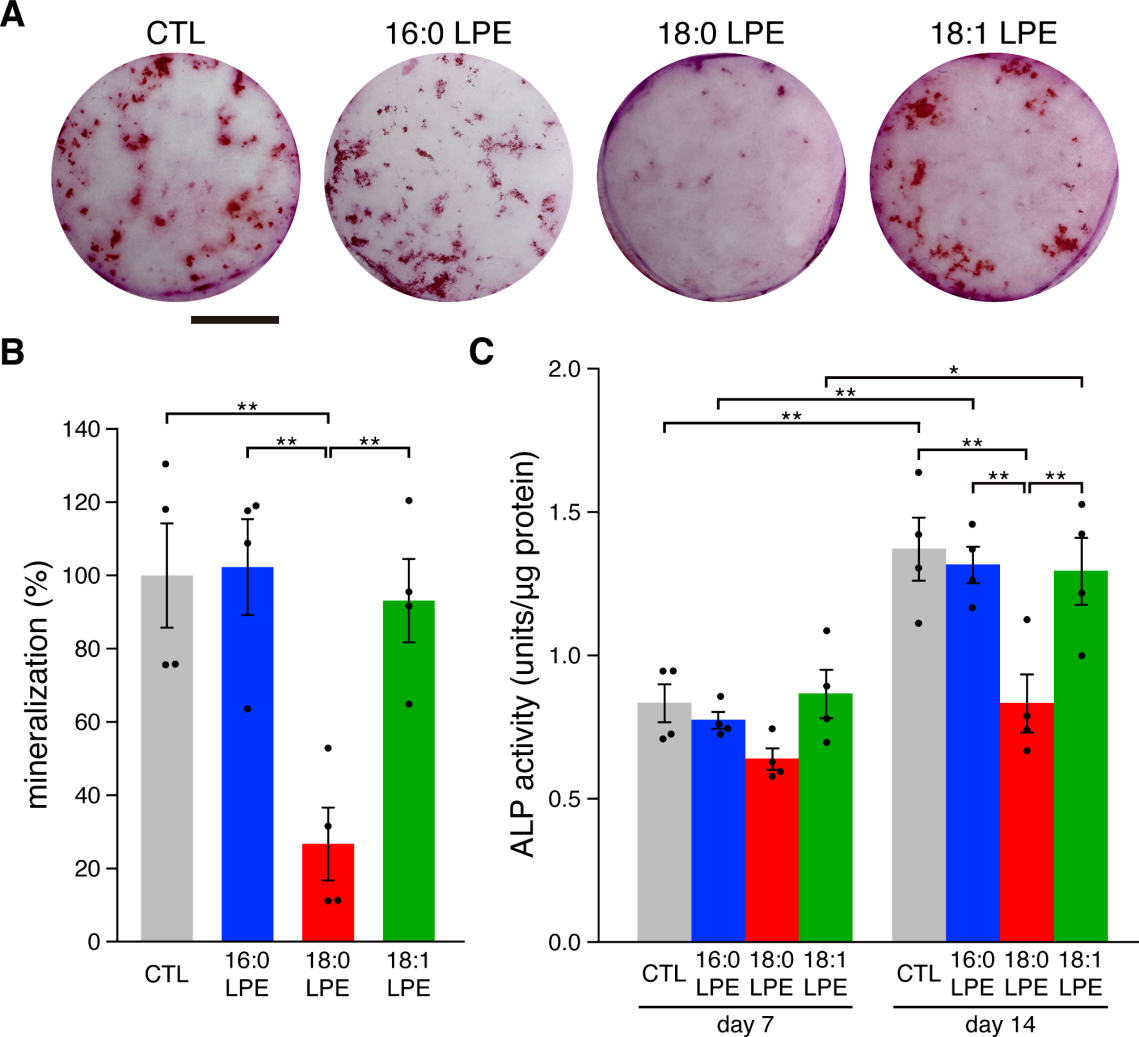
Differential effects of LPE species on MC3T3-E1 cell osteogenic differentiation. (A) Effects of 16:0 LPE, 18:0 LPE, and 18:1 LPE on mineralized nodule formation in MC3T3-E1 cells. Cells were cultured in an osteogenic induction medium for 25 days in the presence or absence of 20 µM LPEs. Mineralized nodules were stained with Alizarin Red S. Scale bar, 5 mm. (B) Quantification of mineralization by measuring the mineralized nodule area in (A). Data are presented as a percentage of the control. (C) ALP activity in MC3T3-E1 cells. MC3T3-E1 cells were cultured in the presence or absence of LPEs for 7 and 14 days, and ALP activity was quantified. Data are presented as mean ± SEM (*n* = 4). **p < 0.01 and *p < 0.05; one-way ANOVA followed by a post-hoc Tukey’s test.

Osteogenic differentiation is a characteristic cellular response in MC3T3-E1 cells. MC3T3-E1 cells were cultured in an osteogenic induction medium with or without LPEs. After 25 days, cells were stained with Alizarin Red S to assess the level of mineralized nodule formation. Mineralized nodules were observed in both the control culture plate and the culture plates treated with 16:0 LPE or 18:1 LPE (Fig. 5A), with no difference in the mineralized nodule area. However, that area was significantly reduced in cells treated with 18:0 LPE (Fig. 5B), suggesting the suppression of mineralization. Next, alkaline phosphatase (ALP) activities were measured to further examine the effects of LPEs. ALP activity increased in MC3T3-E1 cells from day 7 to 14 in the control, 16:0 LPE- and 18:1 LPE-treated cultures (Fig. 5C), with no significant differences among the groups. In contrast, 18:0 LPE-treated cultures did not exhibit increased ALP levels.

## Differential effects of structurally distinct LPE species on osteogenic gene expression in MC3T3-E1 cells

**Fig. 6:**
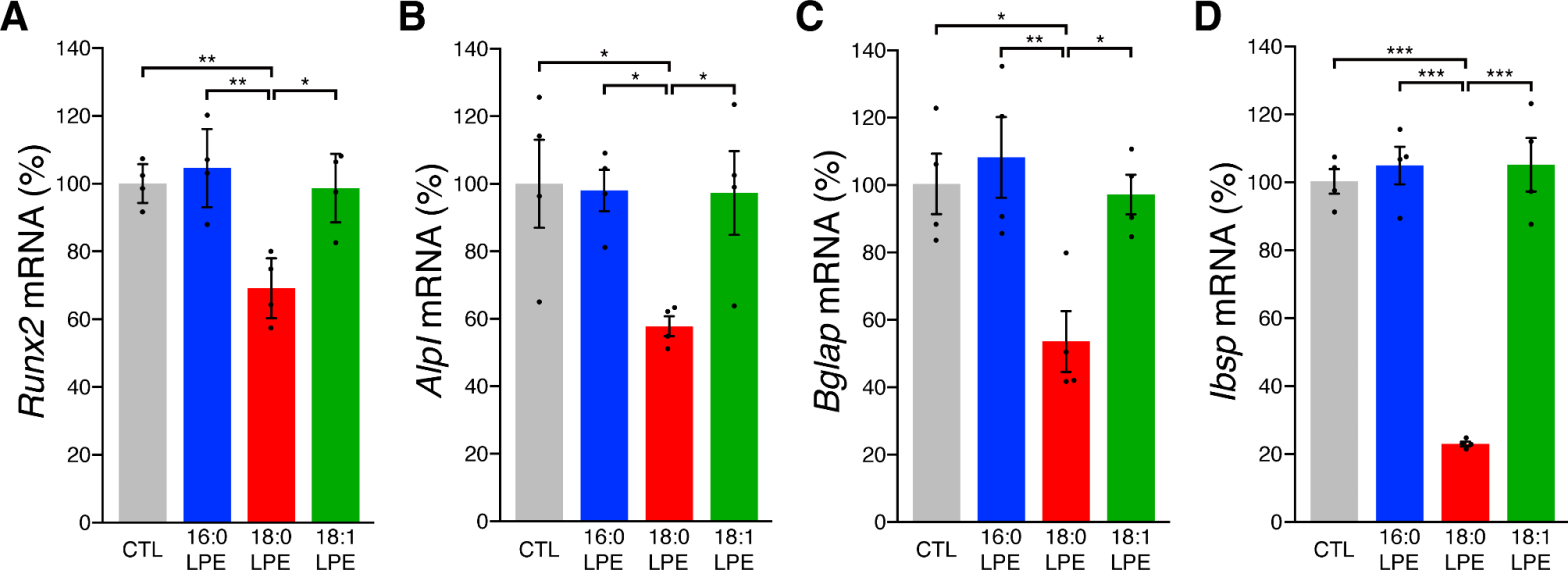
Differential effects of LPE species on osteogenic gene expression in MC3T3-E1 cells. (A–D) Quantitative real-time PCR analysis of gene expression in MC3T3-E1 cells. Cells were cultured in an osteogenic induction medium supplemented with or without 20 µM 16:0 LPE, 18:0 LPE, or 18:1 LPE, and *Runx2* (A), *Alpl* (B), *Bglap* (C), and *Ibsp* (D) mRNA expressions were analyzed by quantitative real-time PCR. *Runx2* mRNA was analyzed one day after incubation, while other genes were analyzed after 14 days. mRNA levels of each gene were normalized to those of *Gapdh*. Data are presented as mean ± SEM (*n* = 4). ***p < 0.001, **p < 0.01, and *p < 0.05; one-way ANOVA followed by post hoc Tukey’s test.

To assess the effects of LPE species on MC3T3-E1 cell differentiation at the transcriptional level, we analyzed the expression of genes associated with osteoblast differentiation. One day after osteogenic induction, we examined the *Runx2* transcript, one of the earliest markers of osteoblast differentiation^14^. In 18:0 LPE-treated cells, the level of *Runx2* mRNA was significantly lower than in the control culture (Fig. 6A). 18:0 LPE-treated cells also displayed a significant reduction in *Alpl*,* Bglap*, and *Ibsp* mRNA levels 14 days after osteogenic induction compared with the control (Fig. 6B–D). In contrast, no differences in osteogenic gene levels were observed among the control, 16:0 LPE-treated cells, and 18:1 LPE-treated cells.

## Discussion

**Fig. 7:**
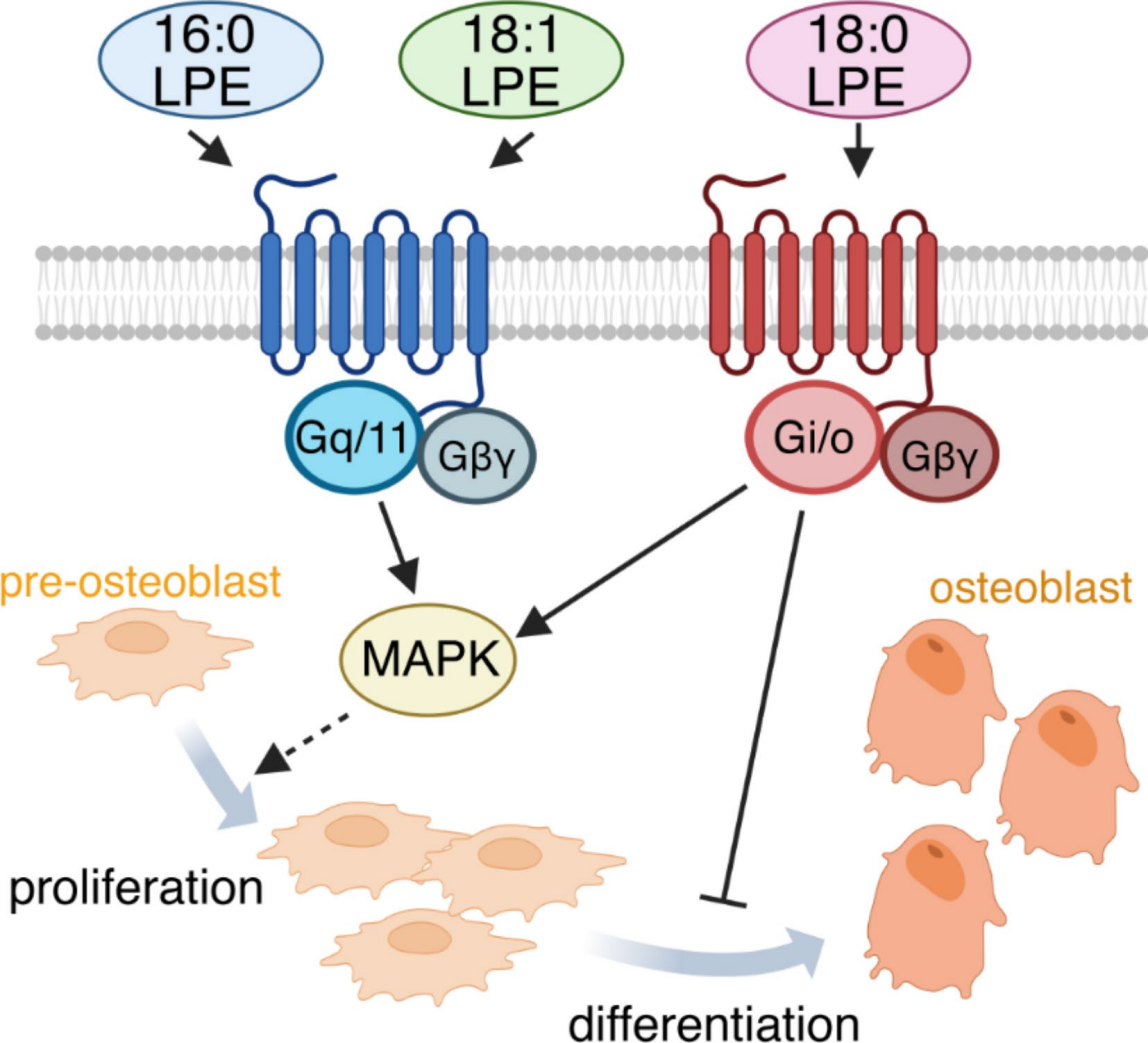
Model depicting the differential effects of LPE molecular species on pre-osteoblast cellular processes. 16:0 LPE and 18:1 LPE promote pre-osteoblast proliferation through the activation of Gq/11-coupled GPCRs that are inhibited by YM-254890. Created with BioRender.com. In contrast, 18:0 LPE promotes pre-osteoblast proliferation while inhibiting their differentiation through PTX-sensitive Gi/o proteins-coupled GPCRs.

LPEs play an important role in various cellular responses, although their effects vary across cell types. However, the impact of LPEs on bone formation remains to be elucidated. This study demonstrated that different LPE species distinctly affect the proliferation and differentiation of pre-osteoblast MC3T3-E1 cells by interacting with distinct GPCRs (Fig. 7).

Structurally different LPE species, 16:0 LPE, 18:0 LPE, and 18:1 LPE, promote the MC3T3-E1 cell proliferation (Fig. 1). Furthermore, these LPEs stimulated the MAPK/ERK1/2 pathway (Fig. 2), whose activation often correlates with increased cell proliferation^26^. Upon stimulation with 18:0 LPE, the phosphorylation of MAPK/ERK1/2 peaked at 10 min. On the other hand, stimulation with 16:0 LPE and 18:1 LPE resulted in the peak phosphorylation of MAPK/ERK1/2 at 60 min (Fig. 2A–C). Thus, the activation of MAPK/ERK1/2 by 16:0 LPE and 18:1 LPE was markedly slower than the rapid response observed in many cells. Such delayed activation of MAPK/ERK1/2 in MC3T3-E1 cells has been reported in multiple studies^27–30^, although the mechanism remains unclear. Further studies are needed to clarify this phenomenon.

Importantly, the MAPK/ERK1/2 activation was mediated by different GPCR types. Inhibitor experiments suggest that 16:0 LPE and 18:1 LPE activate MAPK/ERK1/2 via Gq/11-coupled GPCRs, whereas 18:0 LPE acts via Gi/o-coupled GPCRs (Fig. 2D–F).This differentiation aligns with the Ca^2+^ responses observed (see Fig. 3); 16:0 LPE- and 18:1 LPE-treated MC3T3-E1 cells displayed Ca^2+^mobilization typical of the phospholipase C pathway linked to Gq/11-coupled receptors^31^. In contrast, 18:0 LPE, acting via Gi/o-coupled GPCRs, did not elicit Ca^2+^ responses, aligning with the notion that the activation of Gi-coupled receptors does not necessarily lead to Ca^2+^mobilization^32,33^. The receptor specificity of LPE is consistent with our previous findings in cultured primary cortical neurons; 16:0 LPE and 18:1 LPE function through Gq/11-coupled GPCRs, whereas 18:0 LPE operates via Gi/o-coupled GPCR^10,11^. Furthermore, in cultured cortical neurons, LPE promotes neurite outgrowth and protects the cells from glutamate-induced toxicity^10,11^. Contrary to our results, some studies have reported that 18:1 LPE activates signals via PTX-sensitive Gi/o-coupled GPCRs in human neuroblastoma SH-SY5Y cells, human breast cancer MDA-MB-231 cells, human ovarian cancer SK-OV3 cells, and rat pheochromocytoma PC 12 cells^8,21,23,24^. Thus, it appears that LPEs may activate different GPCR types depending on the cell type. Some GPCRs interact exclusively with one specific type of G protein, whereas other GPCRs couple to a diverse array of G protein families, depending on the cell type or the specific ligand involved^31^.

The results from the receptor desensitization experiment suggest that 16:0 LPE and 18:1 LPE act through distinct GPCRs to mediate calcium responses in MC3T3-E1 cells (Fig. 4). Additionally, our results suggest that both 16:0 LPE and 18:1 LPE exert their effects via GPCRs distinct from LPA1 receptor (Fig. 4). The receptors for LPEs remain poorly identified; however, several studies suggest the presence of cell type-specific GPCRs for various LPE species^8,21–24^. For example, in MDA-MB-231 cells, 18:1 LPE increases [Ca^2+^]i, and this response is inhibited by PTX or the LPA receptor LPA1-selective antagonist, AM095^21,22^. In contrast, in SK-OV3 cells, the increase in [Ca^2+^]i stimulated by 18:1 LPE is blocked by PTX. However, this increase is not affected by the LPA1-selective antagonist AM095 nor by the LPA1- and LPA3-selective antagonists VPC32183 and Ki16425^8,22^. Additionally, MDA-MB-231 cells exhibited [Ca^2+^]i responses to 18:1 LPE but not to 16:0 LPE or 18:0 LPE, indicating variability in receptor expression among cells. Similar response specificity to different LPE species has also been observed in other cell types^8,23,24^.

MC3T3-E1 cell mineralization and differentiation was inhibited by 18:0 LPE (Figs. 5 and 6), which interacts with PTX-sensitive Gi/o protein-coupled GPCRs (Fig. 2). Extensive research has been conducted on the role of GPCRs in osteoblast differentiation and mineralization. These studies have revealed that various signaling pathways from G proteins, such as Gαs, Gαq, and Gαi/o, regulate cellular survival, differentiation, and mineralization^13,15^. Regarding Gi/o signaling, epinephrine has been reported to promote the alkaline phosphatase activity of differentiated MC3T3-E1 cells, with these effects mediated by α1-adrenergic receptors coupled to Gi proteins^34^. Another report demonstrated that the exposure of immature MC3T3-E1 cells to the Gi/o inhibitor PTX reduced F-actin, subsequently leading to the suppression of mineralization at later stages^35^. The activation of the Gi/o-coupled CB2 receptor with a selective agonist has been shown to promote ALP expression and mineralized nodules formation in MC3T3-E1 cells^36^. These reports seem to contradict our finding that 18:0 LPE suppressed the differentiation of MC3T3-E1 cells. In terms of adenylyl cyclase (cAMP) signaling, several studies have demonstrated its positive influence on osteoblast differentiation and mineralization^37–41^; however, some studies have also indicated that cAMP may inhibit this process^42,43^. These differences may be influenced by various factors, such as the developmental stage of the cells, stimulation time, and the involvement of auxiliary signaling pathways. Additionally, GPCR are not limited to signal transduction mediated solely by G proteins. They also interact with other signaling factors such as arrestin, resulting in highly diverse and complex signaling pathways^44^.

A recent study indicated that 18:1 LPE binds to the transcription factor retinoid-related orphan receptor gamma t (RORγt), promoting the differentiation of T helper 17 (T_H_17) cells induced by RORγt and their pathogenicity^45^. Therefore, it is also possible that the actions of LPE on MC3T3-E1 may be mediated by mechanisms other than GPCR signaling. Identifying and analyzing GPCR for LPEs would solve this problem.

Our study demonstrates that LPEs act as important mediators in regulating bone formation. Specifically, we found that LPEs selectively promote the proliferation of pre-osteoblast MC3T3-E1 cells while concurrently inhibiting their differentiation, and these effects depend on the LPE species involved. Our findings provide a foundational framework for further investigations into the roles of LPEs in bone formation. Future research to identify the receptors for each LPE and elucidate their signaling pathways will be crucial for understanding the physiological roles of LPEs in bone formation.

## Materials and methods

### Construction of expression vectors

pPB-CAG.EBNXN^46^and pCMV-hyPBase^47^vectors were kindly gifted from the Sanger Institute. The 1.8-kb *Kpn*I-*Xho*I fragment from pCMV-hyPBase was cloned into the *Kpn*I-*Xho*I site of pCAG-1^48^ to yield pCAG-hyPBase. The Ubc promoter-puromycin expression cassette was amplified by PCR with primers 5′-GGATCCGGCCTCCGCGCCGGGTTTTG-3′ and 5′-GGATCCACACAAAAAACCAACACAC-3′ using pSF-CAG-Ub-Puro (Sigma-Aldrich) as a template and cloned into pGEM-T vector (Promega) to yield pGEM-Ub-Puro. The coding sequence of EGFP was amplified by PCR with primers 5′-AGATCTTCGGTACCGCCACCATGGTGAGCAAGGG-3′ and 5′-CTCGAGTTACTTGTACAGCTCGTCCATGCCG-3′ using pEGFP-C1 (Clontech) as a template and subcloned into the pGEM-T vector to yield pGEM-EGFP. The *Bgl*II-*Xho*I EGFP fragment from pGEM-EGFP and the *Bam*HI fragment containing the Ubc promoter-puromycin expression cassette were cloned into the *Bgl*II-*Xho*I and *Bam*HI sites of pPB-CAG.EBNXN, respectively, to yield pPB-CAG.EGFP-puro. Nuclear localization signals (NLS) were added to the 5′ end of the tdTomato gene using site-directed mutagenesis PCR. The primers used were 5′-GGATCCATGGGACCTAAGAAAAAGAGGAAGGTGATGGTGAGCAAGGGCGAGGAG-3′ and 5′-TAGATCATCGATGCATCTCGAGTTACTTTTACTTGTACAGCTCGTCCAT-3′. The template for this reaction was pCAG-tdTomato^49^ (Addgene plasmid # 83029) and resulted in the construct pCAG-NLS-tdTomato. The coding sequence of NLS-tdTOMATO was amplified by PCR with primers 5′-TTCAGATCTTCGGTACCGCCACCATGGGACCTAAGAAAAAGAG-3′ and 5′-TAGATCATCGATGCATCTCGAGTTACTTTTACTTGTACAGCTCGTCC-3′ using pCAG-nls-tdTomato as a template and cloned into the *Kpn*I-*Xho*I sites of pPB-CAG-EGFP-puro using the GeneArt Seamless Cloning and Assembly Kit (Thermo Fisher Scientific) to yield pPB-CAG-nls-tdTOMATO-puro.

## Cell culture

Pre-osteoblastic MC3T3-E1 cells were obtained from RIKEN Cell Bank (Tsukuba, Japan). MC3T3-E1 cells were cultured as described previously^50^. Briefly, cells were cultured in a maintenance medium comprising α-minimal Essential Medium (α-MEM; Nacalai Tesque) containing 10% fetal bovine serum (Thermo Fisher Scientific) and 100 U/mL Penicillin–Streptomycin (Thermo Fisher Scientific) at 37 °C under a 5% CO_2_ humidified atmosphere.

### Cell growth analysis

An MC3T3-E1 stable cell line expressing EGFP and NLS-tdTOMATO was obtained by co-transfection with pCAG-hyPBase, pPB-CAG-EGFP-puro, and pPB-CAG-nls-tdTOMATO-puro. Subsequently, puromycin-resistant clones were selected at 6 µg/mL. For the cell proliferation experiment, the MC3T3-E1 stable cell line was seeded in 24-well plates at 2,500 cells/well. We used a 24-well plate with a special surface modification (provided by Stella Chemifa Corporation) to culture the cells at a uniform density. One day after seeding, we added 10 µM 16:0 LPE (Echelon Biosciences), 18:0 LPE (Avanti), 18:1 LPE (Cayman), or 18:1 LPA (Tocris Bioscience), each combined with 1 µM autotaxin inhibitor HA130 (Merck Millipore), to the culture medium. In LPA receptor inhibition experiments, 0.5, 1, or 5 µM of the LPA1-selective antagonist AM095 (Cayman Chemical) was added simultaneously. In the cell proliferation experiment, cells were cultured for three days without any medium changes during this period. In this and all subsequent experiments, 16:0 LPE, 18:0 LPE, and 18:1 LPE were prepared in dimethyl sulfoxide at concentrations of 5 mM, 10 mM, and 5 mM, respectively. Subsequently, they were diluted with 0.1% fatty acid-free BSA (Sigma-Aldrich) in water to a concentration of 1 mM. Approximately half of the culture medium was removed, and the LPE solution was gradually introduced into the removed culture medium while thoroughly homogenizing. After complete mixing, the mixture was reintroduced to the original culture. The cells were cultured for three days, and images were captured daily using a fluorescent microscope (Axio Vert.A1, Zeiss) equipped with a CCD camera (AxioCam MRm, Zeiss). Cell density was quantified by counting NLS-tdTOMATO fluorescence using ImageJ software version 1.53a.

### Western blot analysis

MC3T3-E1 cells were seeded in a 6-well plate at a density of 1.0 × 10^5^ cells/well. Following cellular adhesion, the culture medium was replaced with serum-free maintenance medium for a duration of 3–16 h. After serum starvation, cells were pretreated with 1 µM autotaxin inhibitor HA130, either independently or in combination with 5 µM MEK/ERK inhibitor U0126 (Cayman Chemical), 1 µM Gq/11 inhibitor YM-254890 (FUJIFILM Wako Pure Chemical Industries), or 1 ng/mL Gi/o inhibitor PTX (FUJIFILM Wako Pure Chemical Industries) for 30 min at 37 °C. Subsequently, 10 µM 16:0 LPE, 18:0 LPE, or 18:1 LPE was added. The concentration of 10 µM was determined based on observations that, when cells were stimulated with 18:1 LPE, phosphorylated MAPK/ERK1/2 was detected starting at 5 µM, with an increase observed up to 20 µM (Supplementary Fig. 3). To ensure consistency with the cell proliferation experiments, all LPE concentrations were standardized to 10 µM. After incubation for 0–120 min at 37 °C, the cells were harvested in ice-cold PBS and lysed in 20 µL RIPA buffer containing 50 mM Tris-HCl (pH 8.0), 150 mM NaCl, 1% NP-40, 0.5% DOC, 0.1% SDS, 1 mM EDTA, protease inhibitor (Roche), and phosphatase inhibitor (Nacalai Tesque) for 10 min on ice. After sonication, the samples were centrifuged at 20,400 × *g* for 30 min. The supernatants were boiled in SDS sample buffer, separated by SDS-PAGE, and transferred to a PVDF membrane. The membranes were blocked using PVDF Blocking Reagent for Can Get Signal (TOYOBO) and incubated with mouse anti-phospho-p44/42 MAPK (Erk1/2) (Thr202/Tyr204) antibody (1:1000; clone E10, Cell Signaling Technology), followed by incubation with horseradish peroxidase (HRP)-conjugated secondary antibody. After stripping, the membranes were incubated with rabbit anti-p44/42 MAPK (Erk1/2) antibody (1:1000; Cell Signaling Technology), followed by HRP-conjugated secondary antibody. Proteins were visualized using an ECL Select Western Blotting Detection System (GE Healthcare) and detected using a Las-4000 mini luminescent imaging analyzer (GE Healthcare).

### Ca^2+^ imaging

Intracellular Ca^2+^ concentration was measured using Fura 2-AM (Dojindo). MC3T3-E1 cells were seeded at 1.0 × 10^4^ cells/well in a clear-bottomed 96-well plate. After 1 day of incubation, cells were loaded with 10 µM Fura 2-AM for 45 min at 37 °C in a physiological salt solution (PSS) containing 0.05% Pluronic F-127 (Sigma-Aldrich). PSS comprised the following: 115 mM NaCl, 5.4 mM KCl, 0.8 mM MgCl_2_, 1.8 mM CaCl_2_, 13.8 mM glucose, and 20 mM HEPES (pH 7.4). Before imaging, the loading solution was replaced with 50 µL of PSS containing autotaxin inhibitor HA130 and incubated for 30 min. Subsequently, 50 µL of PSS containing 16:0 LPE, 18:0 LPE, 18:1 LPE, or 18:1 LPA was added to achieve a final concentration of 10 µM, and images were captured at 2-second intervals. The analysis was conducted using PSS containing 1 µM of the autotaxin inhibitor HA130 throughout the entire experiment. In certain experiments, an additional 1 µM of the Gq/11 inhibitor YM-254890 was included to assess its effects. During the desensitization experiments, a second administration was performed 60 s after the start of measurement. For this step, 50 µL of PSS containing LPEs or 18:1 LPA was added, ensuring a final concentration of 10 µM. The images were captured using a fluorescence microscope (AxioObserver Z1, Zeiss) equipped with a 10× objective lens (EC Plan-Neofluar 10×/0.3 Ph1, Zeiss) and a CCD camera (Axiocam 702 mono, Zeiss). The images were taken with an emission of 510 nm and excitation of 340 nm and 380 nm. The fluorescence emission ratio at the two excitation wavelengths (340/380 nm) was evaluated as an indicator of intracellular Ca^2+^ concentration. The images were analyzed and visualized using ImageJ Fiji (Version 2.9.0/1.53t).

### Mineralization assay

The mineralization assay was performed essentially as described previously^50^. Briefly, MC3T3-E1 cells were seeded in 24-well plates at 3.0 × 10^4^ cells/well and cultured in a maintenance medium until confluence. Cell differentiation was induced in an osteogenic induction medium containing 20 µM of 16:0 LPE, 18:0 LPE, or 18:1 LPE. This medium consists of a maintenance medium supplemented with 5 mM β-glycerophosphate (Merck Millipore) and 100 µg/mL L-ascorbic acid (FUJIFILM Wako Pure Chemical Industries). Half of the osteogenic induction medium was replaced every 3 days with a fresh osteogenic induction medium containing LPE. After 25 days, cells were stained using a calcified nodule Staining kit (Cosmo Bio) according to the manufacturer’s instructions. Briefly, cells were washed three times with phosphate-buffered saline and fixed with pre-chilled methanol for 20 min. After washing with water three times, cells were stained with Alizarin Red S for 5 min. Images were captured using a microscope equipped with a digital camera (Tough TG-5, OLYMPUS). The percentage of the mineralized area was measured using ImageJ software 1.53q.

### Alkaline phosphatase activity assay

After 7 or 14 days of incubation with an osteogenic induction medium containing 20 µM of 16:0 LPE, 18:0 LPE, or 18:1 LPE, cells were washed three times with saline and lysed with 1% NP-40 in saline. Protein concentration was measured using a Protein Assay BCA Kit (Nakalai Tesque). Alkaline phosphatase activity was measured using the LabAssay ALP kit (FUJIFILM Wako Pure Chemical Industries) according to the manufacturer’s instructions. The values were expressed as units per microgram of protein. One unit of ALP activity is the release of 1 nmol of p-Nitrophenol per minute at pH 9.8 and 37 °C under the conditions described in the manufacturer’s instructions.

### PCR analysis

For quantitative real-time PCR analysis, total RNA was isolated from MC3T3-E1 cells using TRIzol Reagent (Thermo Fisher Scientific) after culture in osteogenic induction medium for 1 and 14 days. ReverTra Ace qPCR RT Master Mix (TOYOBO) was used to synthesize first-strand cDNA. Quantitative RT-PCR assays were performed using the QuantStudio 3D Digital PCR System (Thermo Fisher Scientific) with a TaqMan Fast Advanced Master Mix for qPCR. The TaqMan probes used were *Runx2* (assay ID, Mm00501584_m1), *Alpl* (assay ID, Mm00475834_m1), *Ibsp* (assay ID, Mm00492555_m1), *Bglap* (assay ID, Mm03413826_mH), and *Gapdh* (assay ID, Mm99999915_g1) for normalization of gene expression. *Runx2* mRNA was analyzed one day after incubation with the osteogenic induction medium, while other genes were analyzed after 14 days of incubation.

For semi-quantitative PCR analysis of *Enpp2 mRNA*, which encodes autotaxin, total RNA was extracted using TRI Reagent (Molecular Research Center) from the adipose tissue of ICR mice, which is known for high Enpp2 mRNA expression^51^, and from MC3T3-E1 cells. First-strand cDNA synthesis was carried out using the ReverTra Ace qPCR RT Master Mix. PCR amplification was performed with the following primers: 5′-GCCCTGATGTCCGTGTATCT-3′ and 5′-CGTTTGAAGGCAGGGTACAT-3′ for *Enpp2*, and 5′- CATGGCCTTCCGTGTTCCTA-3′ and 5′-GCGGCACGTCAGATCCA-3′ for *Gapdh*. The reactions were conducted using KOD FX Neo polymerase (TOYOBO) under the following conditions: one cycle of 94 °C for 2 min, followed by 30, 35, and 40 cycles of 98 °C for 10 s, 55 °C for 30 s, and 68 °C for 30 s. The animal care and experimental protocols were reviewed by the Committee for Animal Experiments and approved by the president of Shinshu University (Authorization No. 024010).

### Statistical analysis

The results of at least two independent experiments were subjected to statistical analyses. Statistical significance was evaluated using two-way repeated ANOVA or one-way ANOVA followed by Tukey’s or Dunnett’s post hoc test, and Student’s t-test using R software (R Core Team, 2017). Data are presented as mean ± SEM. Statistical significance was assumed when *p* < 0.05.

## Electronic supplementary material

Below is the link to the electronic supplementary material.

Supplementary Material 1

## Data Availability

The data generated in this study are available from the corresponding author upon reasonable request.
