# Supplementary material for: Differential effects of structurally different lysophosphatidylethanolamine species on proliferation and differentiation in pre-osteoblast MC3T3-E1 cells
Source: Sci Rep. 2025 Jan 2;15:466. doi: 10.1038/s41598-024-84176-8 (PMC11696160; doi:10.1038/s41598-024-84176-8)

Supplementary Information

Differential effects of structurally different lysophosphatidylethanolamine species on proliferation and differentiation in pre-osteoblast MC3T3-E1 cells

Fumiaki Makiyama[1,2], Shiori Kawase[3], Aoi William Omi[2], Yusuke Tanikawa[1,2], Taishi Kotani[4], Teruki Shirayama[1,2], Naoyuki Nishimura[5], Taiga Kurihara[6,7], Naoto Saito[5], Jun Takahashi[1,2], Takeshi Uemura[2,3,4,5*]

[1]Department of Orthopedic Surgery, Shinshu University School of Medicine, Nagano 390-8621, Japan

[2]Department of Biomedical Engineering, Graduate School of Medicine, Science and Technology, Shinshu University, Nagano 390-8621, Japan

[3]Division of Gene Research, Research Center for Advanced Science and Technology, Shinshu University, Nagano 390-8621, Japan

[4]Department of Biomedical Engineering, Graduate School of Science and Technology, Shinshu University, Nagano 390-8621, Japan

[5]Institute for Biomedical Sciences, Interdisciplinary Cluster for Cutting Edge Research, Shinshu University, Nagano 390-8621, Japan

[6]Division of Microbiology and Molecular Cell Biology, Nihon Pharmaceutical University, Saitama 362-0806, Japan

[7]Division of Physiology, Faculty of Medicine, Saga University, Saga 849-8501, Japan

Correspondence and requests for materials should be addressed to T.U. (email: tuemura@shinshu-u.ac.jp)

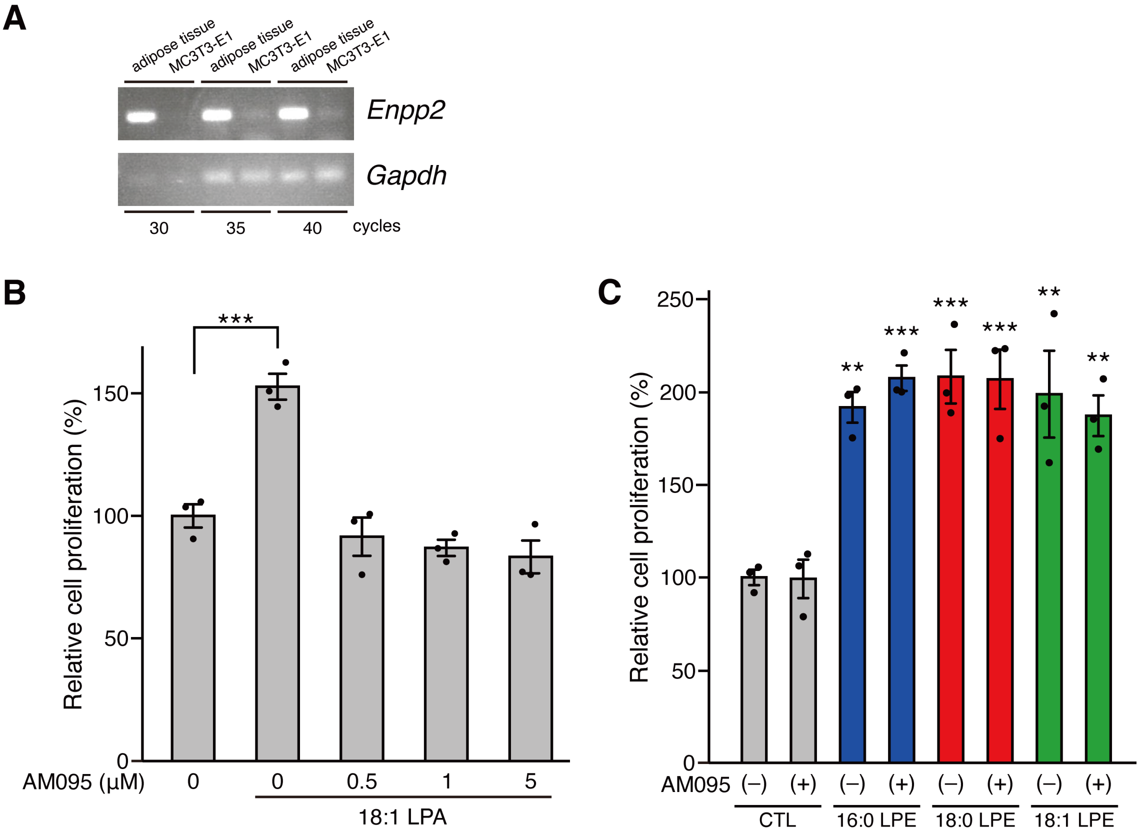

Supplementary Figure 1. Effects of LPA1 antagonist AM095 on LPE-promoted MC3T3-E1 cell proliferation. (A) Semi-quantitative PCR analysis of *Enpp2* mRNA expression in MC3T3-E1 cells. Total RNA was extracted from MC3T3-E1 cells and adipose tissue, the latter of which expresses high levels of *Enpp2* mRNA encoding autotaxin, and was analyzed by PCR using *Enpp2*- and *Gapdh*-specific primers. Agarose gel electrophoresis images of the fragments amplified by PCR are shown. (B) Effect of 18:1 LPA on cell proliferation and the impact of LPA1 inhibitor AM095. 18:1 LPA at a concentration of 10 µM was added to the cultures with varying concentrations of AM095. After three days of incubation, the number of cells was quantified. (C) Effects of AM095 on the proliferation of 16:0 LPE, 18:0 LPE, and 18:1 LPE. Each LPE at a concentration of 10 µM was added to cell cultures, with or without 1 µM AM095, and the cell numbers were measured after three days. Data are presented as mean ± SEM ($n = 3$). ***$p < 0.001$ and **$p < 0.01$ compared to control without AM095; one-way ANOVA, followed by a post-hoc Tukey's test.

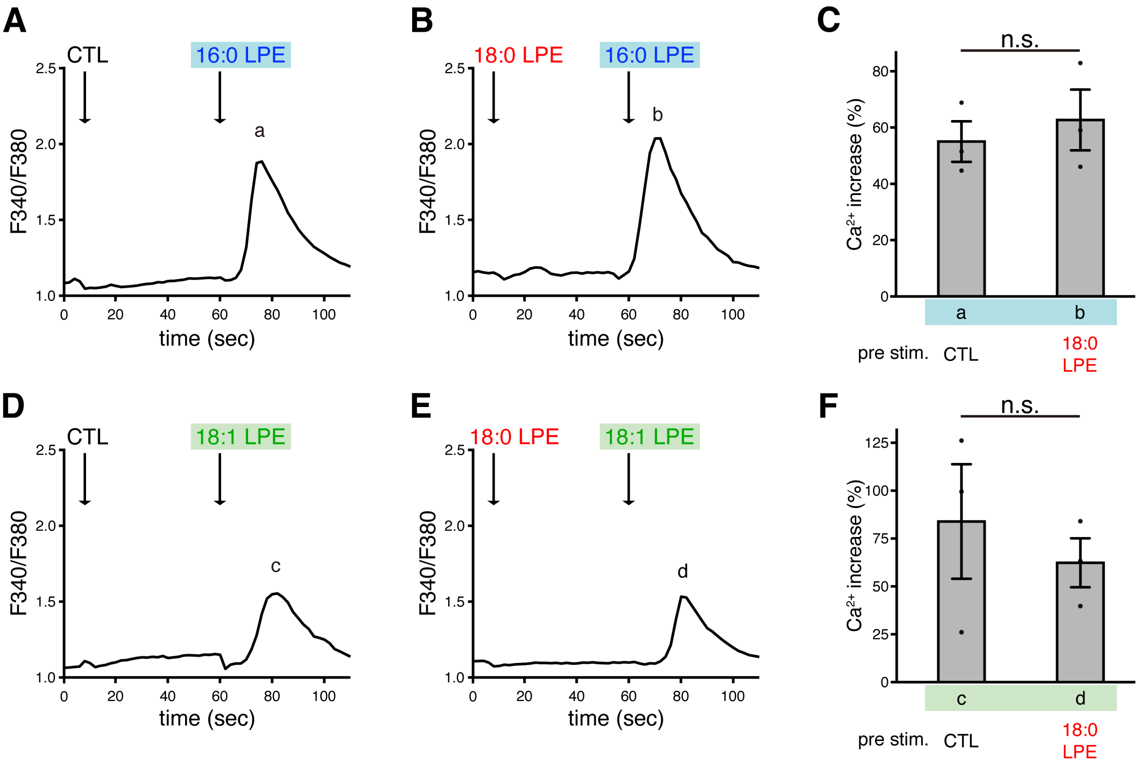

Supplementary Figure 2. Effects of pre-stimulation of 18:0 LPE on 16:0 LPE- and 18:1 LPE-induced Ca^{2+} responses in MC3T3-E1 cells. (A, B) Effects of pre-stimulation with 18:0 LPE on the 16:0 LPE-induced Ca^{2+} response. Cells were pre-stimulated with either the control vehicle (CTL; DMSO) or 18:0 LPE prior to stimulation with 16:0 LPE. Changes in the F340/F380 ratio, an indicator of $[Ca^{2+}]i$, are shown. (C) Summary of the results in (A) and (B). The maximum percentage increase in intracellular calcium levels $[Ca^{2+}]i$ was quantified for comparison. (D, E) Effects of pre-stimulation with 18:0 LPE on the 18:1 LPE-induced Ca^{2+} response. Cells were pre-stimulated with either the control vehicle (CTL; DMSO) or 18:0 LPE prior to stimulation with 18:1 LPE. Changes in the F340/F380 ratio are shown. (F) Summary of the results in (D) and (E). The maximum percentage increase in $[Ca^{2+}]i$ was quantified. Data are presented as mean ± SEM (n = 3). In all experiments, 16:0 LPE, 18:0 LPE, and 18:1 LPE were added at a concentration of 10 μM.

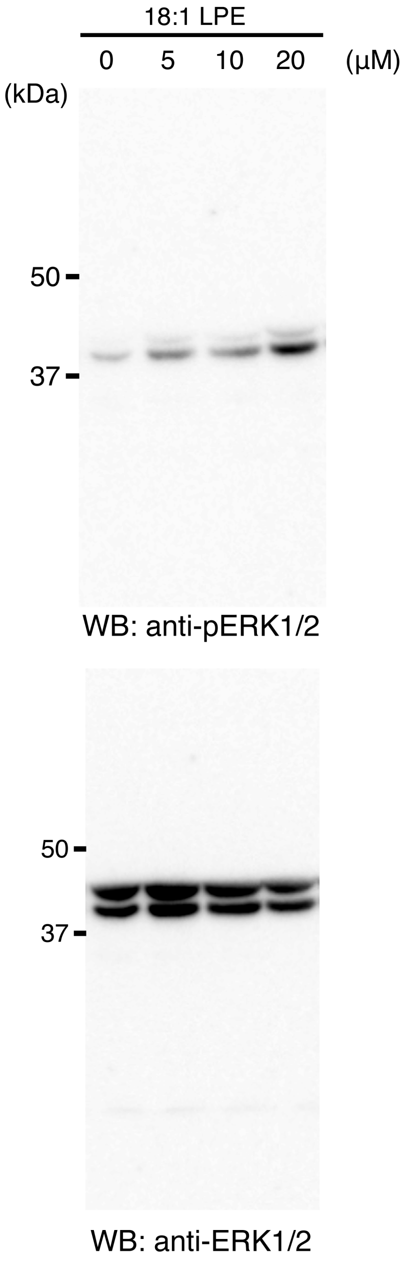

Supplementary Figure 3. Dose-dependent effects of LPE on MAPK/ERK1/2 activation. MC3T3-E1 cells were stimulated with 5, 10, and 20 μM 18:1 LPE for 5 min. After incubation, cells were lysed and analyzed by western blotting using antibodies against ERK1/2 and pERK1/2 proteins.

Supplementary Figure 4. Original images of blots shown in Fig. 2.

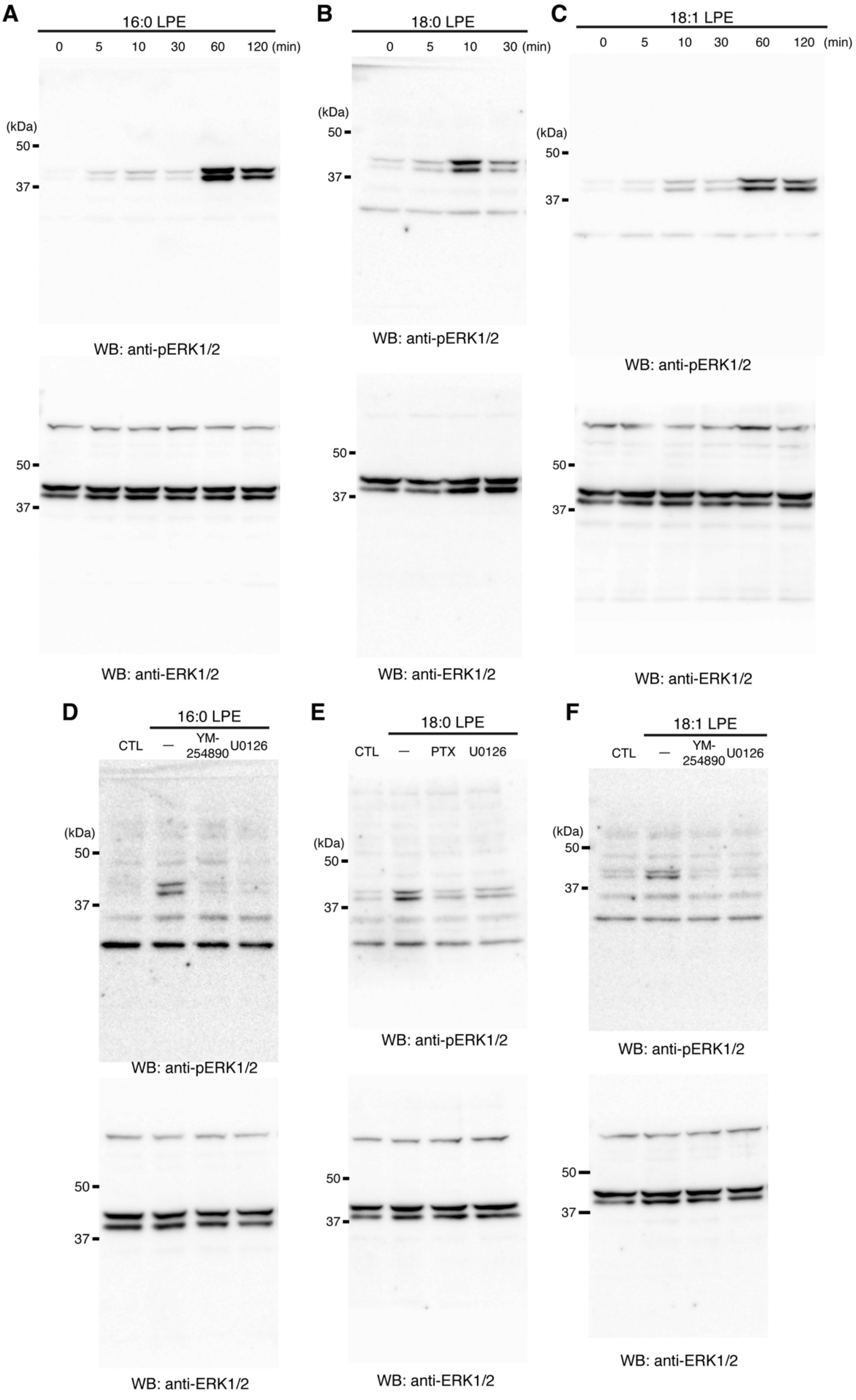

Supplement: Supplementary file 1 — Supplementary Material 1 [file 41598_2024_84176_MOESM1_ESM.pdf]
